# Atomistic characterization of the maturation mechanisms in the HIV-1 capsid domain

**DOI:** 10.1038/s41467-026-71988-7

**Published:** 2026-04-16

**Authors:** Caroline Cannistra, Ethan Wang, Elizabeth M. Y. Lee, Alvin Yu

**Affiliations:** 1 Department of Physiology and Biophysics, University of California, Irvine, Irvine, CA USA; 2 Center for Virus Research, University of California, Irvine, Irvine, CA USA; 3 Center for Complex Biological Systems, University of California, Irvine, Irvine, CA USA; 4 Department of Materials Science and Engineering, University of California, Irvine, Irvine, CA USA; 5 Department of Chemical and Biomolecular Engineering, University of California, Irvine, Irvine, CA USA

**Keywords:** Computational biophysics, Molecular dynamics, Retrovirus

## Abstract

During HIV-1 maturation, capsid proteins (CA) are proteolytically cleaved from the immature Gag lattice and undergo conformational transitions in which the amino-terminal and carboxy-terminal domains rearrange into mature conformations prior to assembling into fullerene complexes. Little is known, however, of the structural transitions and energetic principles governing this transformation. Here, we reveal the dynamical mechanisms of CA maturation using all-atom and enhanced sampling simulations. Free energy calculations demonstrate that immature CA is intrinsically unstable as a monomer and rapidly converts into a mature form that is consistent with x-ray crystallographic and cryo-electron microscopy structures. Analysis of the minimum free energy pathway indicates that conformational change in CA proceeds through stepwise, ratcheting transitions, and we identify a metastable intermediate distinct from the immature and mature endpoints. Energy decomposition analysis uncovers the interdomain residue-residue interactions, particularly charged contacts, that stabilize the mature state. These results reveal the interactions and molecular mechanisms that drive the conformational changes required for HIV-1 capsid domain maturation.

## Abstract (web summary)

Atomistic simulations reveal how HIV-1 capsid proteins transition from immature to mature forms. By identifying the molecular pathway, a metastable intermediate, and energetics, these findings highlight the stepwise conformational changes and key interactions that stabilize the mature capsid.

## Introduction

HIV-1 maturation is a crucial stage of the viral life cycle where immature, spherical Gag lattices within virions disassemble and reassemble into fullerene cone-shaped capsids that transport viral genetic material into the host cell nucleus. The capsid protein (CA) starts as a domain within the Gag polyprotein, and cleavage of Gag by viral protease releases CA, which assembles into a lattice of hexamers and pentamers that form the mature viral capsid^1–7^. Each CA monomer is composed of an amino-terminal domain (NTD) and a carboxy-terminal domain (CTD)^8^. Maturation involves large-scale conformational changes in CA, including NTD-CTD reorientation and the remodeling of inter-subunit contacts^9–12^. The resulting fullerene capsids are composed exclusively of CA in these rearranged, mature conformations, stabilized by newly formed cross-domain contacts and the loss of others^10^. These conformational transitions highlight the remarkable plasticity of CA, which enables both capsid assembly and makes it vulnerable to therapeutic intervention. Indeed, the success of lenacapavir, a first-in-class long-acting capsid inhibitor, demonstrates that CA is a clinically tractable antiviral target^13,14^.

Despite these advancements, the molecular mechanisms driving the conformational transitions required for HIV-1 CA maturation remain incompletely understood. Structural studies have elucidated CA organization within immature and mature lattices, identifying hexameric and pentameric assemblies across tubular and conical morphologies, as well as higher-order lattice structures^5,6,9,15^. Both in vitro assembly and molecular simulations demonstrated that inositol phosphates (IP_6_) significantly extend the stable lifetimes of mature capsids, illustrating how small molecules modulate lattice energetics^16,17^. Furthermore, free energy simulations reveal that lattice stability within the mature capsid depends on hexamer and pentamer packing and curvature, which can be modulated by interactions with molecules like IP_6_ and the PF74 inhibitor^18^. Atomistic simulations of the complete mature capsid have provided insight into mechanical strain during uncoating^19^, and enhanced sampling simulations have been used to examine the energetics of IP_6_ binding^17^ and metabolite import^20^. Although coarse-grained approaches have shown that the immune sensor protein TRIM5α cages the mature capsid and that capsid shape regulates transport across the nuclear pore^21,22^, current coarse-grained models lack the resolution to capture the immature-to-mature transition. Similarly, structural techniques such as x-ray crystallography and cryo-electron microscopy (cryo-EM) are unable to capture the transient, heterogeneous intermediates that bridge the immature and mature states^23,24^. While nuclear magnetic resonance (NMR) spectroscopy experiments have provided evidence of CTD dimerization, reorientation, and linker contraction in CA, suggesting that these processes are central to maturation^25^, the precise mechanisms that underlie maturation remain unresolved.

In this study, we map the maturation pathway of HIV-1 CA using all-atom molecular dynamics simulations. Enhanced sampling simulations reveal the intrinsic free energy landscape underlying conformational changes in CA associated with NTD-CTD reorientation, and path-sampling techniques identify the minimum free energy pathway (MFEP) linking the immature and mature conformations. These simulations show that the immature conformation is unstable and uncover a previously uncharacterized conformational intermediate during maturation. Using end-point energy decomposition analysis, we identify the network of residue interactions that stabilize distinct conformational states along the MFEP. In particular, we find that sequentially engaging and disengaging NTD–CTD contacts promote the stepwise progression from immature to mature states. Together, these results provide an atomic description of the molecular pathway underlying HIV-1 CA domain maturation.

## Results

### The free energy landscapes of CA conformational change

**Fig. 1:**
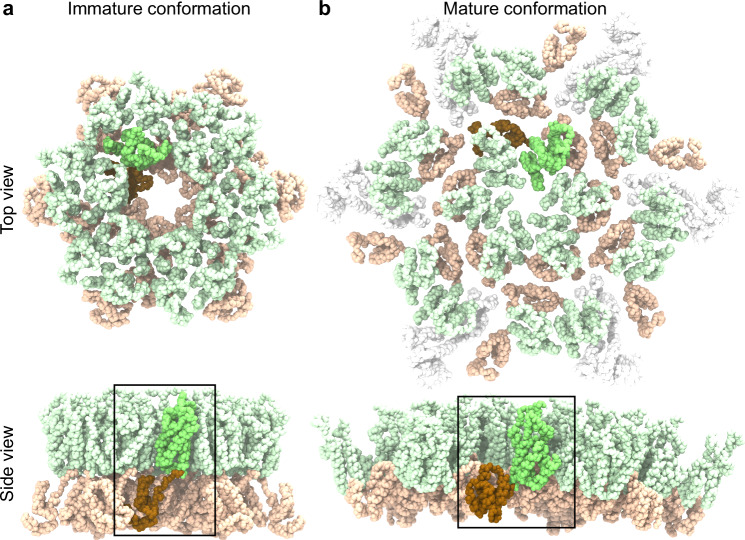
Structures of the HIV-1 capsid lattice. **a** In the immature virion, the capsid lattice forms a hexagonal array in which each hexamer is tightly bundled with trimeric contacts between neighboring hexamers (PDB ID: 4USN). **b** Upon maturation, conformational changes in CA resulting in the mature capsid lead to lattice expansion, with an increase in surface area of approximately $872\,{{{{\AA }}}}^{2}$ per monomer (PDB ID: 5MCX). In both panels, the NTD is shown in green, and the CTD is shown in brown. 18 CA monomers are shown. Six additional monomers of the mature lattice are shown in white. A single CA monomer is highlighted in each lattice. Top and side views are shown for both lattices to highlight differences in curvature.

In cryo-EM structures of immature virions^9^, CA is tightly packed into spherical Gag lattices, which occupy a surface area of 719 ${{{\mathrm{\AA}}} }^{2}$ per CA monomer (Fig. 1a). Upon maturation, CA undergoes a conformational change in which the CTD twists relative to the NTD, resulting in an expansion to 1591 ${{{\mathrm{\AA}}} }^{2}$ per monomer^16^ (Fig. 1b). Structurally, interactions at the CTD-CTD interface within an immature hexamer and interactions at the NTD-NTD interface across immature hexamers are lost to form the mature capsid. Disassembly of the immature Gag lattice after proteolytic cleavage thus involves the dissociation of CA, which are present in solution typically as monomers or dimers^25^. In atomic-force microscopy images of mature capsid formation, individual monomers, dimers, and higher-order oligomers were detected to associate and dissociate from the growing capsid lattice^26^. As the monomer is the minimal biological unit capable of the conformational changes required for maturation, and because the monomer is not constrained by lattice contacts or interfacial interactions so it samples the broadest conformational space, we modeled the conformational changes required for maturation in a CA monomer. To determine the energetics of the transition, we computed the free energy landscape, i.e., the potential of mean force (PMF), of conformational change using an umbrella sampling strategy^27^. The atomic model for the monomer was extracted from the cryo-EM structure of a mature CA hexamer (PDB ID: 6BHT). Similarly, a monomer from a CA hexamer of the immature Gag protein was used as a reference configuration of the immature state (PDB ID: 4USN). NMR spectroscopic measurements have demonstrated that CA forms dimers across an interface located within the CTD, which does not prevent the relative rotations of the NTD-CTD required for maturation^25,28^, indicating that the conformational changes and associated energetics of a CA dimer are similar to those of two separate monomers.

**Fig. 2:**
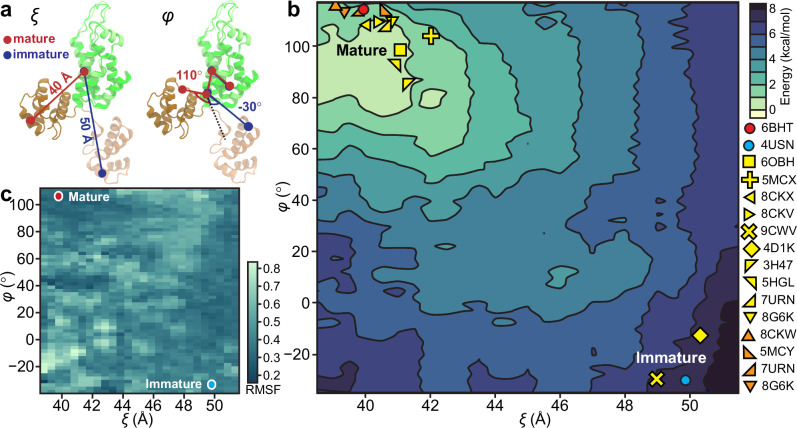
The free energy landscape of the molecular maturation of CA protein. **a** Two order parameters ($\xi$ and $\varphi$) were used to characterize the conformational change in CA during maturation. $\xi$ is defined as the distance between the centers-of-mass of residues in helix 7 of the NTD and in helix 10 of the CTD. The dihedral parameter $\varphi$ is defined by the centers-of-mass of four residue groups in: helices 1 and 2, the N-terminal of helix 7, the C-terminal of helix 7, and the linker between helices 8 and 9, to quantify the rotational motion of the CTD relative to the NTD. See Methods for a complete description. The immature conformation is semi-transparent, whereas the mature conformation is opaque. **b** The free energy landscapes for CA maturation plotted as a 2D PMF (potential of mean force). Contour lines correspond to energy differences of 1 kcal/mol. The location of cryo-EM and crystal structures of CA in the hexamer (in either mature or immature conformations) are indicated by yellow markers. Structures of CA in pentamers are shown with orange markers. Blue and red dots correspond to the immature and mature conformation of CA (PDB ID: 4USN and 6BHT, respectively). **c** The root-mean-square fluctuation (RMSF) of the linker region backbone across the order parameter space. RMSF values range from 0-1 Å. Source data are provided as a Source Data file.

A two-dimensional order parameter $(\xi,\varphi )$ was used to quantify CA conformational change from the immature to mature, in which $\xi$ is defined as the distance between the centers-of-mass of  two selections of amino acid residues in the NTD and CTD respectively, and $\varphi$ is defined as a torsional angle between the NTD, helix 7, and the CTD (Fig. 2a). Steered molecular dynamics simulations were performed to generate the initial configurations for umbrella sampling. In aggregate, a total of 47.1 *μ*s of trajectory statistics was collected and reweighted using the Weighted Histogram Analysis Method (WHAM)^29^.

Interestingly, the free energy landscape revealed a sharp energetic minimum around the mature state of CA, with a global free energy minimum at (39 Å, 90°) (Fig. 2b). Immature configurations of the CA protein were destabilized relative to the mature state by approximately 8 kcal/mol. In addition to the global minimum at the mature state, the PMF also identifies two locally stable intermediate CA configurations that were occupied during the immature-to-mature transition. One intermediate positioned at (42 Å, 80°), was proximal to the global minimum corresponding to the mature CA conformation, and the other was a metastable state positioned at (46 Å, 10°), between the mature and immature conformations. Comparison of available structures of CA in the Protein Data Bank showed that the structures were localized within 1–2 kcal/mol of the CA conformation that was occupied in the structure, indicating that there was good agreement between the available experimental structures and the ensemble of immature and mature conformations. Pentamers had slightly higher dihedral values and lower distances (${\xi }_{{{\rm{pen}}}}=39-41{{\mathrm{\AA}}},{\xi }_{{{\rm{hex}}}}=40-43{{\mathrm{\AA}}},{\varphi }_{{{\rm{pen}}}}=110-115^\circ,{\varphi }_{{{\rm{hex}}}}=85-110^\circ$), indicating that pentameric monomers had a greater twist in the relative orientations of the NTD and CTD, and were slightly more compact than hexamers. These results suggest that isolated CA monomers are not stable in the immature state, and instead rapidly transition to the mature conformation upon CA-SP1 cleavage and dissociation. This behavior is consistent with biochemical reconstitutions of maturation in vitro, in which viral protease digestion of Gag triggers the conversion of immature-like virus particles to mature capsid-like particles^30^ and the immature-to-mature transition primarily reflects the loss of constraints at MA-CA contacts, the CA-SP1 junction, and inter-hexamer interactions.

We also measured the root-mean-square fluctuation (RMSF) of the CA NTD-CTD linker to assess whether there was a significant disorder-to-order transition in the linker that occurred as the CA protein transitioned from the immature to mature state. The RMSF of the linker was measured for the ensemble of conformations corresponding to each $(\xi,\varphi )$ value (Fig. 2c). Although fluctuations of the linker in the mature state and in the immature state were low (0.32 ${{\mathrm{\AA}}}$ and 0.36 ${{\mathrm{\AA}}}$, respectively) there was a small, but noticeable increase in the fluctuations of the linker at intermediate states of maturation (0.65 ${{\mathrm{\AA}}}$). The fluctuation differences were <1 ${{\mathrm{\AA}}}$, suggesting that the linker does not play a dominant role in the conformational change required for maturation.

### The minimum free energy pathway for CA maturation and intermediate states

To identify the most probable transition pathway, we used the string method^31–33^, a chain-of-states approach in which transition states between the immature and mature end configurations are iteratively optimized to find a physical pathway for CA maturation or minimum free energy pathway (MFEP). Briefly, the free energy landscape or 2D PMF is interpolated using Gaussian process regression. An initial pathway of transition states is generated by linearly interpolating between the two end configurations. Each transition state is then shifted by a specified drift magnitude along the PMF gradient, computed using a first-order finite difference. The pathway is then reparametrized to maintain an equal distance between the transition states in the order parameter space. The shift of transition states and pathway reparameterization are iteratively repeated until the pathway converges to the MFEP.

**Fig. 3:**
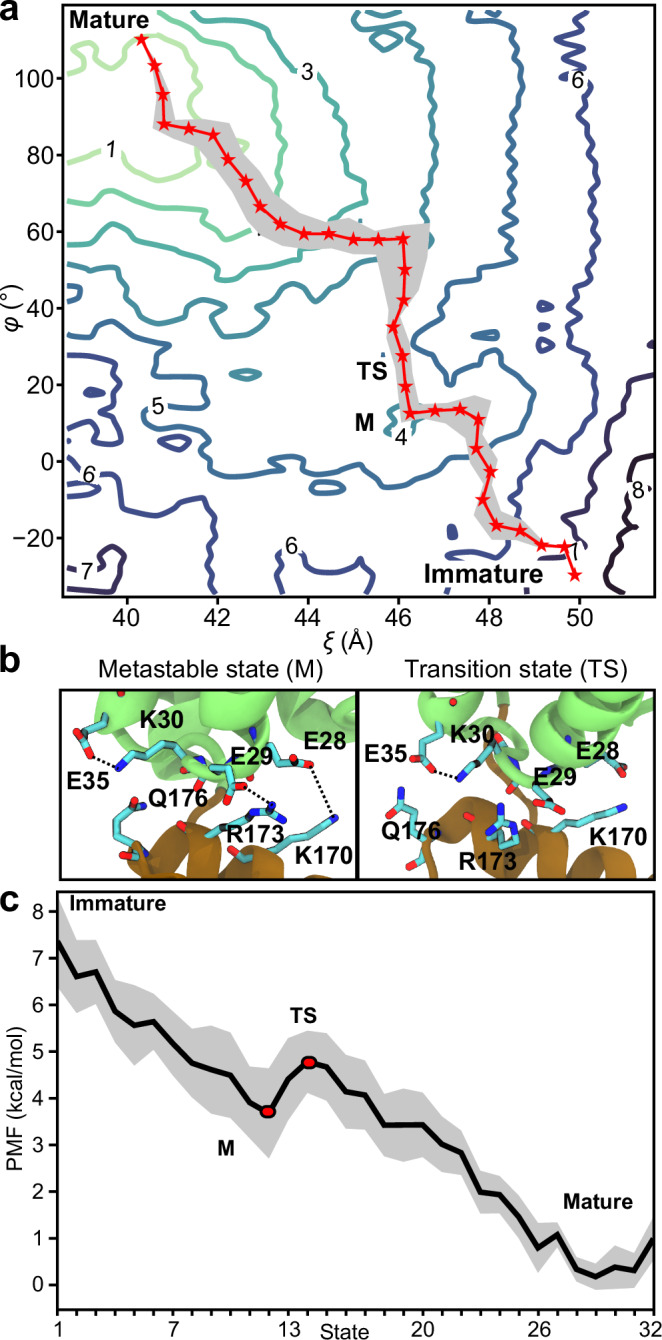
Conformational intermediates in HIV-1 capsid maturation. **a** The minimum free energy pathway (MFEP). The final converged pathway derived from 60 ns of trajectory data, marking each of the 32 identified states (red line). Gray shaded regions denote the standard deviation of pathway coordinates measured from the pathway in six 10 ns blocks of trajectory data. The immature state, the mature state, a metastable state (M), and a transition state (TS) are labeled. Contour lines correspond to energy differences of 1 kcal/mol. **b** The CA conformation corresponding to the metastable and transition states. The metastable state is stabilized by interactions between E28-K170 and E29-R173, while the transition state is stabilized by K30-R173 and E29-K170 interactions. **c** The 1D PMF along the MFEP across the state index (black line; 1 corresponds to immature, 32 corresponds to mature). Red dots mark key intermediates. Shaded regions indicate the standard deviation of the PMF across six 10 ns blocks of trajectory data. Source data are provided as a Source Data file.

Application of the string method to CA maturation revealed that capsid maturation proceeds in a stepwise fashion. In the immature conformation, the separation between the NTD and CTD initially decreases from $\xi=50{{\mathrm{\AA}}}$ to $\xi=48\,{{\mathrm{\AA}}}$, followed by a twisting of the relative orientation of the NTD and CTD $\varphi=-20^\circ$ to $\varphi=10^\circ$, (Fig. 3a). Subsequent compactification of the distance between the NTD and CTD brings the CA monomer to a metastable local energetic minimum at $(\xi,\varphi )=(46\,{{\mathrm{\AA}}},\,10^\circ )$, where the metastable conformation is stabilized by electrostatic interactions across the NTD and CTD between K170–E28, R173–E29, and K30–E35–Q176 (Fig. 3b). These transient interactions break as the CA monomer shifts to an unstable transition state, with the E29-R173 bond breaking and R173 re-positioning to disrupt the K30-E35-Q176 interaction, as the NTD and CTD rotate from $\varphi=10^\circ$ to $\varphi=60^\circ$. Concomitant shortening of the NTD and CTD distance ($\xi$) and re-orientation of the NTD and CTD from $(\xi,\,\varphi )=(46\,{{\mathrm{\AA}}},\,60^\circ )$ to $(41\,{{\mathrm{\AA}}},\,85^\circ )$, followed by a rotation of the NTD and CTD about the dihedral $\varphi=85^\circ$ to $\varphi=110^\circ$ transitions the CA monomer into the mature conformation. The highest standard deviation on any coordinate from block averaging across six blocks of 10 ns trajectories was $(0.5\,{{\mathrm{\AA}}},\,6^\circ )$.

Analysis of the free energy profile along the MFEP (Fig. 3c) showed a slightly rugged free energy landscape consistent with a stepwise mechanism for maturation, in which alternating compactification and rotation of the NTD and CTD in the monomer are stabilized by amino acid residue interactions across the NTD and CTD interface. Standard error in the PMF was computed with a block averaging approach using six blocks of 10 ns each. The free energy profile for the maturation pathway is primarily downhill, except for a barrier at the transition state, which is ~1kcal/mol higher than the nearby metastable state, indicating that these two conformations are key stages in the conformational change during maturation. Overall, the free energy difference between the mature and immature state is ~7.3 kcal/mol.

### The molecular mechanism of maturation and end-point free energy calculations

**Fig. 4:**
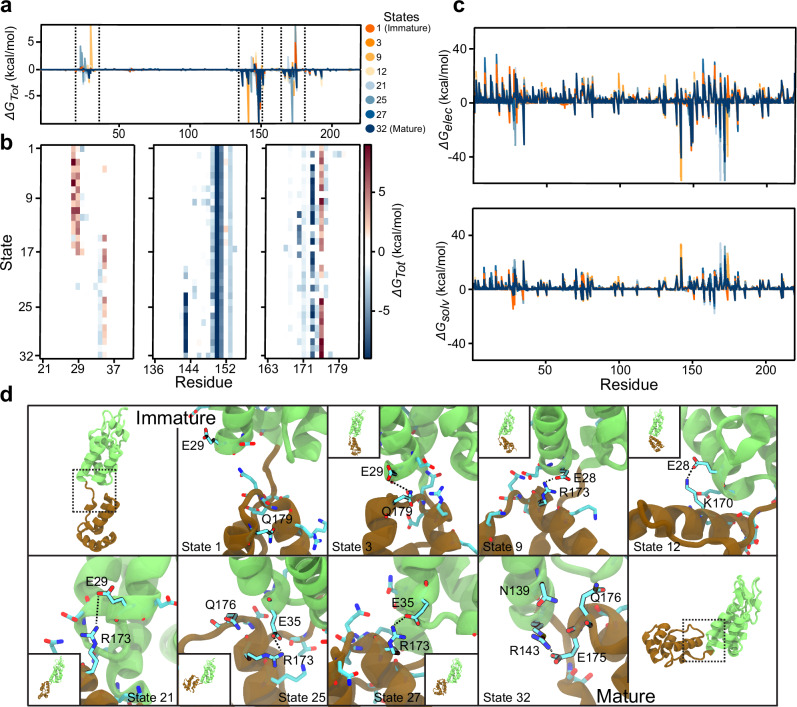
Free energy decomposition and mechanism of the maturation pathway. **a** The total binding free energy $(\triangle {G}_{{Tot}})$ along the maturation MFEP is computed using molecular mechanics Poisson–Boltzmann surface area (MM/PBSA) methods and decomposed on a per-residue basis. Dotted lines highlight the three regions with the highest contributions to the total free energy across the pathway. Each color corresponds to a specific conformational state along the immature-to-mature transition, progressing from orange (immature) to blue (mature). **b** Magnified view of the three regions with the highest per-residue free energy contributions across the MFEP (residues 21–41,136–156, and 163–183), visualized as a heat map. Blue indicates stabilizing (favorable) interactions; red indicates destabilizing (unfavorable) contributions. **c** The binding free energy decomposed into electrostatic $(\triangle {G}_{{elec}})$ and solvation $(\triangle {G}_{{solv}})$ free energies. **d** Snapshots of the molecular maturation of CA. Dashed lines indicate the most stable interaction pairs. Residue pairs in each transition state corresponding to the most energetically favorable pairwise contacts are highlighted. In the immature state, contact between E29-Q179 quickly forms at state 3. In state 9, E28 forms a salt bridge interaction with R173. K170 displaces R173 to interact with E28 and then switches interaction partners from E28 to E29 at state 12, the metastable state. Interactions between R173 and E35 persist as the CTD rotates in $\varphi$ from $10^\circ$ to $70^\circ$ (states 12-25). Additional salt bridge contacts form between R143 and E175, and Q176 interacts with N139 as the CTD shifts into the mature conformation (states 25 to mature). Source data are provided as a Source Data file.

To quantify the energetic contributions of contact formation between amino acid residues in the maturation pathway, we performed end-point free energy calculations using the Molecular Mechanics Poisson Boltzmann Surface Area (MM/PBSA) method in which a thermodynamic cycle is used to compute the relative free energy change between two states by estimating the solvation free energy of two configurations, the gas phase free energy change between the two states, and the entropic contributions from quasi-harmonic approximations^34^. End-point free energy calculations were performed for each state along the MFEP. The analysis was carried out treating the NTD (residues 1–150) and CTD (residues 151–221) as distinct molecular groups. Consistent with umbrella sampling results, there was a progressive decrease in total free energy as CA transitioned from the immature to the mature conformation (Fig. 4a, Supplementary Movie S1). Per-residue decomposition of the free energy revealed that the greatest change in binding free energy occurred in three distinct regions of CA, residues 21–41, 136–156, and 163–183, at the NTD-CTD interface. Close-up views of individual amino acid residue contributions to free energy as CA transitioned from the immature to the mature conformation showed that the process was highly dynamic, with individual residues either stabilizing or destabilizing CA at progressive stages of maturation (Fig. 4b).

Further decomposition of the total free energy into electrostatic interactions, van der Waals forces, and solvation effects revealed that, interestingly, hydrophobic (or van der Waals) contacts between the NTD and CTD (Supplementary Fig. 2) contributed much less strongly to maturation. Instead, maturation is primarily driven by electrostatic interactions across the NTD-CTD interface. For residues outside the three regions, enthalpic contributions to the free energy from electrostatic interactions were counter-balanced by a loss of entropy in solvation. For residues within the three regions, electrostatic interactions provide strong net stabilization contributing the strongest to the free energy change at each state of the pathway (Fig. 4c, Supplementary Movie 1).

The sequence of structural transitions that characterizes the MFEP for CA maturation is as follows (Fig. 4d, Supplementary Movie 2). In the immature state, there are relatively few contacts between the NTD and CTD. Instead, the CA monomer is largely stabilized by contact with adjacent monomers in the lattice. Once the CA monomer dissociates from the immature lattice, contact between Q179 and E29 shifts the CTD closer to the NTD (state 1–3). Initially, transient interactions form between R173 and E28, driving a relative rotation of the CTD (state 9). K170 then shifts to contact E28, displacing R173 (state 12). R173 then interacts with E29 as the CTD settles into a kinked conformation in which the axes of helices 1 and 8 are nearly orthogonal (state 21). The guanidino group of R173 then switches to interact with the carboxyl sidechain of E35, which also contacts the carbonyl oxygen of Q176 (state 25). Contact between K170 and E28 are lost as the CTD rotates backward about the NTD-CTD linker, stabilized by contact between R173 and E35 in a pre-mature conformation (state 27). Finally, a salt bridge forms between R143 and E175, and Q176 shifts to interact with N139 as the CTD rotates into the mature conformation (state 32). Notably, I150 and L151 residues in the NTD-CTD linker regions were identified as strongly contributing to maturation, whereas the adjacent S149 and T148 did not, consistent with mutagenesis experiments^35^.

To assess the energetic contributions to the free energy landscape of maturation of each amino acid pair contact, we also performed a pairwise decomposition using MM/PBSA for each state along the maturation pathway (Supplementary Movie 3, Supplementary Fig. 3). Residue-residue interactions that stabilized the secondary structure of the protein, in this case, primarily helices, were readily apparent in the pairwise interaction maps as expected, likely owing to the i and i + 4 hydrogen bonding patterns of $\alpha$-helices. However, the largest changes in pairwise interaction energy appear in the off-diagonal elements, corresponding to NTD-CTD contacts involving the same three regions identified above (residues 21–41, 136–156, 163–183). Analysis of these residue-residue contacts supports a model in which progressive maturation is promoted by the formation of specific electrostatic interactions at the NTD-CTD interface.

## Discussion

Collectively, our all-atom molecular dynamics simulations and free energy calculations reveal that CA undergoes a conformational transition during maturation mediated by a series of electrostatic contacts between the NTD and CTD. These simulations quantified the free energy landscape of CA conformations and identified an MFEP that contains a metastable intermediate state. We find that the immature CA conformation is intrinsically unstable and is instead stabilized by the packing of the Gag lattice, inter-hexamer NTD-NTD contacts, and intra-hexamer CTD-CTD contacts^36^. Conversely, pairwise energy decomposition analysis indicates that the mature conformation is stabilized by residues in the NTD helices 1-2 and CTD helix 8 that form salt bridges, hydrogen bonds, and van der Waals interactions in addition to intra-hexamer NTD-NTD contacts and inter-hexamer CTD-CTD contacts.

Contacts involving E28, E29, E35, K170, and R173 stabilize the transition and metastable states, whereas later intermediates and the mature conformation are supported mainly by E35, R143, R173, and E175. In particular, the E35–R173 salt bridge stabilizes both the mature state and its precursor conformations, consistent with mutagenesis data demonstrating that R173A abolishes viral infectivity^37^. A second salt bridge between R143 and E175 also contributes to mature-state stabilization, though its role appears less critical as R143A mutants remain viable^38^. Mutational studies reinforce the role of E28/E29, K30, K170, and E175 in capsid assembly or infectivity^23,37,38^, while Q176 and E35 remain uncharacterized. Overall, these findings reveal a hierarchy of residue interactions (e.g., E35-R173) that are critical for maturation.

Our analysis of the NTD-CTD linker suggests that it undergoes only minor fluctuations during the conformational transition, indicating that the linker likely does not play a major role in driving the conformational change. These results rule out the hypothesis that the linker region switches between a disordered and ordered state during maturation. Instead, our findings suggest that the linker region contributes indirectly – for example, by facilitating the formation of higher-order CA oligomeric states, including dimers, pentamers, or hexamers – and thus plays a more downstream role in lattice assembly. This model is consistent with mutational studies showing that linker variants impair capsid assembly, core stability, and viral infectivity^35,39–41^.

From a therapeutic standpoint, reinforcing stabilizing contacts of the immature or metastable conformations could trap CA in nonfunctional states, blocking infectivity, while strategies aimed at destabilizing the mature conformation may similarly disrupt the viral life cycle. Recent cryo-EM structures show that the small-molecule lenacapavir (LEN) and bevirimat (BVM) bind directly to the immature capsid. While BVM binds to and stabilizes the CA-SP1 junction, LEN engages the NTD interfaces, which can alter the maturation process^42^. Notably, the inhibitor-binding regions for LEN overlap with structural elements of the metastable state – residues R173, M144, and S146 – suggesting that binding restricts the conformational shifts required for maturation. These interactions are expected to increase the free energy barriers between CA conformational states, impeding progression along the maturation pathway. Future studies can investigate how small-molecule therapeutics disrupt the conformational changes and the allosteric networks involved in capsid maturation. Defining these interaction networks may reveal additional structural vulnerabilities that could be exploited to block viral maturation and infectivity.

In vivo, CA maturation occurs within the crowded, highly coordinated macromolecular environment of the HIV virion. This process requires the proteolytic cleavage of the immature Gag lattice, disassembly of the immature CA layer, and the reassembly into a mature, fullerene capsid that encases the viral genome. While the current study uses all-atom simulations to elucidate the conformational changes and mechanisms underlying maturation in individual CA domains—one component of the process—future work to simulate the entire process will require more efficient computational approaches. In this context, atomistically accurate characterization of CA conformations and their intermediates can inform larger-scale, coarse-grained models of HIV-1 virion maturation.

## Methods

### All-atom models for the CA protein

The initial atomic model for the immature HIV capsid protein was constructed from the cryo-EM structure of a hexamer-of-trimers of CA (PDB ID: 4USN^9^). A single CA monomer in the immature conformation was extracted. The initial atomic model for the mature HIV capsid protein was constructed from the x-ray crystallographic structure of a CA hexamer (PDB ID: 6BHT^16^) owing to the 2.9 ${{\mathrm{\AA}}}$ resolution of the complex. A CA monomer in the mature conformation was extracted. Missing amino acid backbones were built using MODELLER^43^ and missing side chains were built using SCWRL4^44^. The mature CA monomer, containing a total of 3454 atoms, was solvated with 18,368 water molecules. Na^+^ and Cl^-^ ions were added to the bulk solution until the salt concentration was 150 mM NaCl to produce an electrostatically neutral system. A buffer of 10 Å between the protein atoms and the boundary of the simulation cell was used. Periodic boundary conditions were imposed on an orthorhombic unit cell of approximately $(87{{\mathrm{\AA}}} \times 87{{\mathrm{\AA}}} \times 87{{\mathrm{\AA}}} )$. The all-atom potential energy function CHARMM36m^45^ for proteins and the TIP3P potential energy function^46^ for water molecules were used.

Similar to prior studies^19,47,48^, the all-atom model of the CA system was energy-minimized using conjugate gradient descent for 10,000 steps and then equilibrated for 300 ps under constant pressure and temperature (NPT) conditions. Simulations in the isothermal–isobaric, NPT, ensemble were performed using a Nosé-Hoover thermostat at 310 K and a Nosé-Hoover-Langevin barostat at 1 atm. Bond lengths for hydrogen atoms were constrained using the RATTLE algorithm^49^. An r-RESPA integrator was used with a timestep of 2 fs; long-range electrostatics were computed every 4 fs^50^. Electrostatic interactions were computed using the particle mesh Ewald algorithm, and short-range nonbonded interactions were truncated to $12\,{{\mathrm{\AA}}}$. All simulations used the all-atom molecular dynamics simulation package NAMD 3^51^.

### Steered molecular dynamics and order parameters

A 2D order parameter $(\xi,\,\varphi )$ was used to describe the conformational changes from the immature to the mature CA protein. Order parameters were selected to distinguish between the end points of the transition and to ensure reversibility of the process^52,53^. Based on the cryo-EM/x-ray crystallographic structures of the immature and mature states (4USN and 6BHT), the immature and mature conformations were distinguished by the relative positions of the NTD and CTD, both in terms of distance between the subdomains and the angular position of the CTD relative to the NTD’s principal axis. Therefore, we selected two order parameters, a distance $\xi$ and a dihedral angle $\varphi$. Several distance and dihedral pairs were evaluated, and the final pair was selected after steered MD simulations, in which biasing forces implemented along the order parameters reproducibly drove the system from the mature state to the immature state with sufficient overlap between adjacent ensembles for reweighting. $\xi$ specifies the center-of-mass distance between residues 131–135 in helix 7 of the NTD and residues 196–203 in helix 10 of the CTD. $\varphi$ is defined as the dihedral angle between the centers-of-masses of residues 16–45 in helices 1 and 2 in the NTD, residues 131–135 in helix 7 of the NTD, residues 136–145 in helix 7 of the NTD, and residues 171–180 in helices 8 and 9 of the CTD, and specifies the rotational motion of the NTD and CTD relative to a line of intersection defined by the central axis of helix 7 which is proximal to the NTD-CTD linker. The 2D order parameter is $(49.8\,{{\mathrm{\AA}}},\,-30^\circ )$ in the immature state and $(40.2\,{{\mathrm{\AA}}},\,110^\circ )$ in the mature conformation.

Steered molecular dynamics was performed using a harmonic biasing potential, ${U}_{\xi }=\frac{1}{2}{k}_{\xi }\,{(\xi -{\xi }_{0})}^{2}$ and ${U}_{\varphi }=\frac{1}{2}{k}_{\varphi }\,{(\varphi -{\varphi }_{0})}^{2}$ on the $(\xi,\,\varphi )$ order parameters with successive simulations slightly shifting the equilibrium distance, ${\xi }_{0}$, in increments of $0.7\,{{\mathrm{\AA}}}$, followed by 100 ps of relaxation in MD. Force constants of ${k}_{\xi }=3$ kcal/mol/${{{\mathrm{\AA}}} }^{2}$ and ${k}_{\varphi }=3 {{\rm{kcal/mol}}}/^{\circ 2}$ were used to maintain the conformation of the protein, with respect to the order parameters. During the steered MD simulations, harmonic restraints were imposed to maintain $\alpha$-helicity in helix 7 (residues 131–145) and helix 6 (residues 111–118) and minimize structural distortions in the N-terminal domain and C-terminal domain. The harmonic biasing potential for both helices was given by ${U}_{a}=\frac{1}{2}{k}_{a}\,{({a}-{a}_{0})}^{2}$, with force constants, ${k}_{a}=10$ kcal/mol maintaining an equilibrium $\alpha$-helical content of,$\,{a}_{0}=0.9$. The $\alpha$-helical content was measured for $N+1$ residues that span ${N}_{0}$ to ${N}_{0}+N$ as:

1 $$	a\left({C}_{\alpha }^{({N}_{0})},{O}^{({N}_{0})},{C}_{\alpha }^{({N}_{0}+1)},{O}^{({N}_{0}+1)},\ldots,{C}_{\alpha }^{({N}_{0}+N)},{O}^{({N}_{0}+N)}\right)\\=	\frac{(1-C_{hb})}{N-1}{\sum }_{n={N}_{0}}^{{N}_{0}+N-2}{{\rm{angf}}}\left({C}_{\alpha }^{(n)},{C}_{\alpha }^{(n+1)},{C}_{\alpha }^{(n+2)}\right) \\ 	+\frac{C_{hb}}{N-3}{\sum }_{n={N}_{0}}^{{N}_{0}+N-4}{{\rm{hbf}}}({O}^{(n)},{N}^{(n+4)})$$

with ${{\rm{C}}}_{{\rm{hb}}} = 0.5$ and where the scoring function for the ${C}_{\alpha }$ backbone (${C}_{\alpha }-{C}_{\alpha }-{C}_{\alpha }$ angle) is given by:

2 $${{\rm{angf}}}\left({C}_{\alpha }^{\left(n\right)},{C}_{\alpha }^{\left(n+1\right)},{C}_{\alpha }^{\left(n+2\right)}\right)=\frac{1-\frac{{\left(\theta \left({C}_{\alpha }^{\left(n\right)},{C}_{\alpha }^{\left(n+1\right)},{C}_{\alpha }^{\left(n+2\right)}\right)-{\theta }_{0}\right)}^{2}}{{\left(\Delta {\theta }_{{tol}}\right)}^{2}}}{1-\frac{{\left(\theta \left({C}_{\alpha }^{\left(n\right)},{C}_{\alpha }^{\left(n+1\right)},{C}_{\alpha }^{\left(n+2\right)}\right)-{\theta }_{0}\right)}^{4}}{{\left(\Delta {\theta }_{{tol}}\right)}^{4}}}$$

with ${\theta }_{0}=88^\circ$, and $\Delta {\theta }_{{tol}}=15^\circ$. The scoring function for the hydrogen bond is given by:

3 $${{\rm{hbf}}}\left({O}^{\left(n\right)},{N}^{\left(n+4\right)}\right)=\frac{1-{\left(\frac{\left|r\left({O}^{\left(n\right)}\right)-r\left({N}^{\left(n+4\right)}\right)\right|}{{d}_{0}}\right)}^{i}}{1-{\left(\frac{\left|r\left({O}^{\left(n\right)}\right)-r\left({N}^{\left(n+4\right)}\right)\right|}{{d}_{0}}\right)}^{j}}$$

with ${d}_{0}=3.3{{\mathrm{\AA}}}$, $i=6$, and $j=8$. Similarly, a second set of simulations were performed in which the equilibrium dihedral angle, ${\varphi }_{0}$, was shifted in increments of $10^\circ$, until the starting state reached the final state, reversibly, to determine an initial transition pathway. Comparison of the final state with the experimentally determined all-atom model revealed the same CA conformation with a root-mean-square deviation (RMSD) difference of 2.0 ${{\mathrm{\AA}}}$.

### Umbrella sampling and free energy landscapes

Initial coordinates for the umbrella sampling windows were obtained using a steered MD approach by varying the equilibrium dihedral angles, ${\varphi }_{0}$, starting from a relaxed state on the initial transition pathway with the same $\xi$ value. Dihedral angles were increased or decreased in $5^\circ$ increments, and subsequently relaxed for 100 ps. The umbrella sampling windows consisted of CA conformations positioned in $(0.7\,{{\mathrm{\AA}}},\,5^\circ )$ increments on a 2D grid of 620 windows, spanning the order parameter space of $\xi=38.1$ to $51.2\,{{\mathrm{\AA}}}$, and $\varphi=-35$ to $115^\circ$.

Production runs were initiated across all windows for 80 ns/window, totaling an aggregate sampling of 47.1 $\mu$s across the order parameter space. Harmonic biasing potentials used for umbrella sampling were ${k}_{\xi }=12$ kcal/mol/${{{\mathrm{\AA}}} }^{2}$ and ${k}_{\varphi }=2 {{\rm{kcal/mol}}}/^{\circ 2}$ centered on $(\xi,\,\varphi )$. Statistics on the $(\xi,\,\varphi )$ order parameters were collected and recorded every 20 fs. The first 20 ns of unequilibrated MD in each window was discarded. Similar to prior studies^17,48,54–56^, the potential of mean force (PMF) with respect to the $(\xi,\,\varphi )$ order parameters was computed using the weighted histogram analysis method to unbias and recombine the sampled distribution functions across all windows^29,57^. A bin size of $(0.5\,{{\mathrm{\AA}}},2.5^\circ )$ was used.

The statistical uncertainty in the PMF was evaluated using block averaging^58^. For each PMF, the $(\xi,\varphi )$ timeseries was divided into 6 blocks. The WHAM was used to compute a PMF from the data in each block, and then the standard deviation was computed. Using 5–15 blocks all gave qualitatively similar results.

### RMSF analysis of the NTD-CTD linker

We computed the root mean square fluctuations (RMSF) of alpha carbon atoms of residues 146 through 150, which make up the linker region between the N-terminal and the C-terminal domains. 1500 positions were sampled across the last 60 nanoseconds to measure the RMSF in each umbrella sampling window, for a total of 93,000 samples. To remove noise from whole-protein movements, we aligned the linker region to a reference structure at each timepoint, then recorded the position of each of the alpha carbons for that timepoint. We also recorded the system’s location in order parameter space at each measured timepoint. After binning the recorded samples using the same 2D grid as the PMF, we found the mean configuration of the linker region within each bin $\bar{x}$ and calculated the RMSF by measuring all deviations from the mean within each bin; ${{\rm{RMSF}}}=\sqrt{\sum {\left({x}_{i}-\bar{x}\right)}^{2}/N}$ where *N* is the number of samples within the selected bin.

### The string method and the minimum free energy path

Gaussian process regression (GPR) was applied to interpolate the PMF and provide a smoothly varying free energy landscape continuous in both the PMF and the first derivative of the PMF. A rational quadratic kernel with length scale $l=0.43$ and scale mixture parameter $\alpha=0.0012$ was used with parameter values selected with the limited-memory quasi-Newton (L-BFGS-B) algorithm implemented in scikit-learn^59–61^.

The string method is a chain-of-states approach that iteratively optimizes the transition states between two end point configurations and has been applied to characterize conformational changes in a biomolecular system^31–33,62,63^. The initial and final states were taken to be the all-atom model of the immature and mature capsid, respectively. An initial transition pathway was constructed between the two states, using a linear interpolation through the order-parameter space between the immature and mature values. A total of 32 images were used to interpolate the transition path. The path was then iteratively refined until convergence to the MFEP as follows: (1) the gradient of the PMF at each image, ${\nabla A}_{i}$, is computed using a first-order finite difference of the GPR-interpolated PMF, (2) each image is moved by a specified drift magnitude, ${v}_{d}=0.001$, along the gradient, and (3) the pathway is reparametrized to maintain an equal distance, defined by the L2 norm, $\parallel \Delta {{\boldsymbol{\zeta }}}\parallel={({\left[\frac{\Delta \xi }{{{{\boldsymbol{\xi }}}}_{{{\boldsymbol{mat}}}{{\boldsymbol{.}}}}-{{{\boldsymbol{\xi }}}}_{{{\boldsymbol{imm}}}{{\boldsymbol{.}}}}}\right]}^{2}+{\left[\frac{\Delta \varphi }{{\varphi }_{{{\boldsymbol{mat}}}{{\boldsymbol{.}}}}-{\varphi }_{{{\boldsymbol{imm}}}{{\boldsymbol{.}}}}}\right]}^{2})}^{1/2}$, between each image, where ${{{\boldsymbol{\zeta }}}}_{{imm}.}=({\xi }_{{imm}.},{\varphi }_{{imm}.})$ is the coordinate of the immature state, and ${{{\boldsymbol{\zeta }}}}_{{mat}.}=\left({\xi }_{{mat}.},{\varphi }_{{mat}.}\right)$ is the coordinate of the mature state. Steps (1) – (3) were repeated until convergence. Convergence was assessed by the sum of the squared distance between the states of the current and prior paths, $x={\sum }_{i}\parallel {{{\boldsymbol{\zeta }}}}_{j+1}-{{{\boldsymbol{\zeta }}}}_{j}\parallel$, for each image, $i$ and iteration, $j$. The paths were considered converged when $x < 0.01$ or at $j=100$ iterations, whichever was first.

### End-point free energies with Molecular Mechanics Poisson Boltzmann calculations

Coordinates for the capsid protein in each transition state were extracted from the MFEP in protein data bank (PDB) format. Each intermediate state was parameterized using the tleap module^64^, loaded with ff14SB^65^ force field^66^, and solvated in a 16 Å TIP3P^46^ water box. Na^+^ and Cl^-^ ions were added to the bulk solution, until the salt concentration was 150 mM NaCl to produce an electrostatically neutral system. Simulations were performed using the GPU-accelerated particle mesh Ewald molecular dynamics (PMEMD) engine^64,67^. Initial energy minimization involved using the steepest descent algorithm for 1,500 steps followed by conjugate gradient for another 1,500 steps. A harmonic restraint of 2.0 kcal/mol/Å² was applied to all atoms of the protein complex during the minimization. MD simulations were equilibrated at first using the canonical ensemble (constant NVT) at 300 K for 50 ps, followed by an additional 250 ps equilibration run at the NPT ensemble with 300 K and 1 atm. A 5 ns of unrestrained production run was performed at the NPT ensemble at 300 K and 1 atm to ensure each structure resides in a locally stable energy minimum and relax force field specific configurations. To maintain an internally consistent energy model, endpoint binding free energies were evaluated using MMPBSA.py, an AMBER-native module, ensuring consistency in the molecular mechanics, Poisson-Boltzmann electrostatic, and nonpolar solvation terms.

End-point binding free energies for each transition state, which was determined using the string method, were computed using the Molecular Mechanics, Poisson-Boltzmann Surface Area (MM/PBSA)^34,68^ tool from the AmberTools23^64,69^ software package. Free energy calculations were conducted via MMPBSA with generalized Born (GB) and Poisson-Boltzmann (PB) implicit solvent models based on explicit solvent MD trajectories^68^. Frames 1-500 from the production trajectory were analyzed at 25 frame intervals resulting in 20 snapshots per complex. Total solvation free energy was computed by the sum of polar, nonpolar, electrostatic, and van der Waals contributions^68,70^. The free energy associated with the conformational switch from state A to state B is given by:

4 $$\Delta {G}_{A\to B,{solvated}}=\Delta {G}_{B,{solvated}}-\Delta {G}_{A,{solvated}}$$

The free energy change for each term is computed by:

5 $$\Delta {G}_{{solvated}}={E}_{{gas}}+\Delta {G}_{{solvation}}-{{TS}}_{{solute}}$$

We can rewrite this expression in terms of the *i*-th frame and the *N*-total number of frames:

6 $$\Delta {G}_{{solvated}}=	 \left[{E}_{{gas}}\right]+\left[\Delta {G}_{{solvation}}\right]-{T}\left[{S}_{{solute}}\right] \\=	 \frac{1}{N}{\sum }_{i=1}^{N}{E}_{i,{gas}}+\frac{1}{N}{\sum }_{i=1}^{N}{\Delta G}_{i,{solvation}}-\frac{T}{N}{\sum }_{i=1}^{N}{S}_{i,{solute}}$$

### Residue pair free energy decompositions

Residue-residue free energy decompositions were computed using the MMPBSA module (idecomp=3) of AmberTools23. This approach computes the binding free energy contribution of residue pairs by considering the solvation free energy on a residue pair-pair basis^68,70^. Pairwise free energy decomposition was performed using the Poisson-Boltzmann (PB) implicit solvent model^68^ applied to solvated MD trajectories generated with TIP3P water. Coordinate files of intermediate structures were used as input for MM/PBSA calculations. Implicit solvent models were applied during post-processing to improve the accuracy of solvation and binding free energy estimates. For each intermediate conformational state, the production run trajectory was sampled at 25-frame intervals, resulting in 20 frames over 5 ns of simulation time. Only residues 1-150 (NTD) and residues 151–221 (CTD) were included in the calculation to avoid same-domain intra-residue contributions. The decomposition analysis was applied to the entire complex, isolating total pairwise contributions while ignoring direct contacts and neighboring residues, enabling mapping of pairwise energetic couplings.

### Reporting summary

Further information on research design is available in the Nature Portfolio Reporting Summary linked to this article.

## Supplementary information

Supplementary Information

Description of Additional Supplementary Files

Supplementary Movie 1

Supplementary Movie 2

Supplementary Movie 3

Reporting Summary

Transparent Peer Review file

## Source data

Source Data

## Data Availability

All data generated and used in this study are available at https://github.com/theory-and-computation/CA-maturation/ or upon request to the corresponding author. Source data for the main figures are provided as a Source Data file. The referenced PDB entries are: 4USN, 5MCX, 6BHT Source data are provided with this paper.

## Code availability

All code generated and used in this study are available at https://github.com/theory-and-computation/CA-maturation/^71^ or upon request to the corresponding author, Alvin Yu.
