# Supplementary material for: Atomistic characterization of the maturation mechanisms in the HIV-1 capsid domain
Source: Nat Commun. 2026 Apr 16;17:5265. doi: 10.1038/s41467-026-71988-7 (PMC13265795; doi:10.1038/s41467-026-71988-7)
Supplement: Supplementary file 1 — Supplementary Information [file 41467_2026_71988_MOESM1_ESM.pdf]

Supplementary Information:

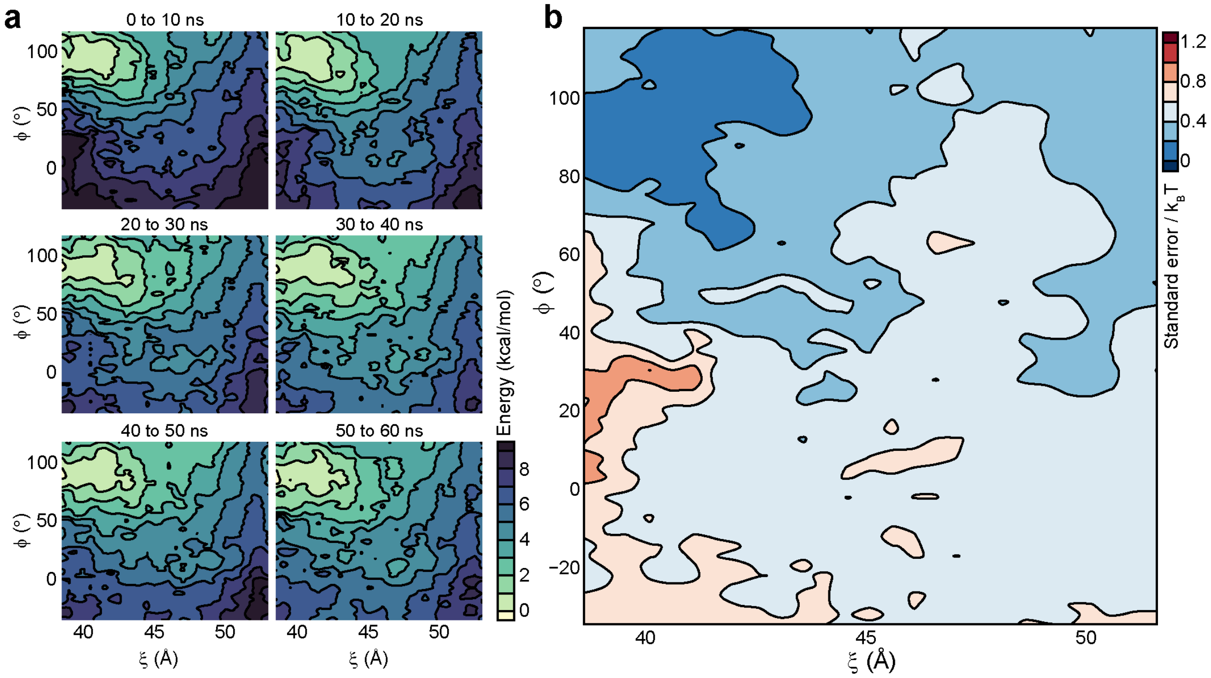

Supplementary Figure 1: Error analysis of the the PMF for CA maturation from block averaging. 60 nanoseconds of umbrella sampling simulations per window were split into blocks of 10 nanoseconds per window each. The Weighted Histogram Analysis Method (WHAM) was applied to compute the PMF for each individual block. **(A)** The PMF of each individual block across CV space. Energy values are in kcal/mol. **(B)** Standard error of the six PMF blocks, computed as the square root of the variance of the PMF in each window divided by the square root of 6 (the number of blocks). The error is given in units of $k_B T$. For a converged PMF, the normalized error is less than 1 and correlated with the PMF with lower error in lower energy regions.

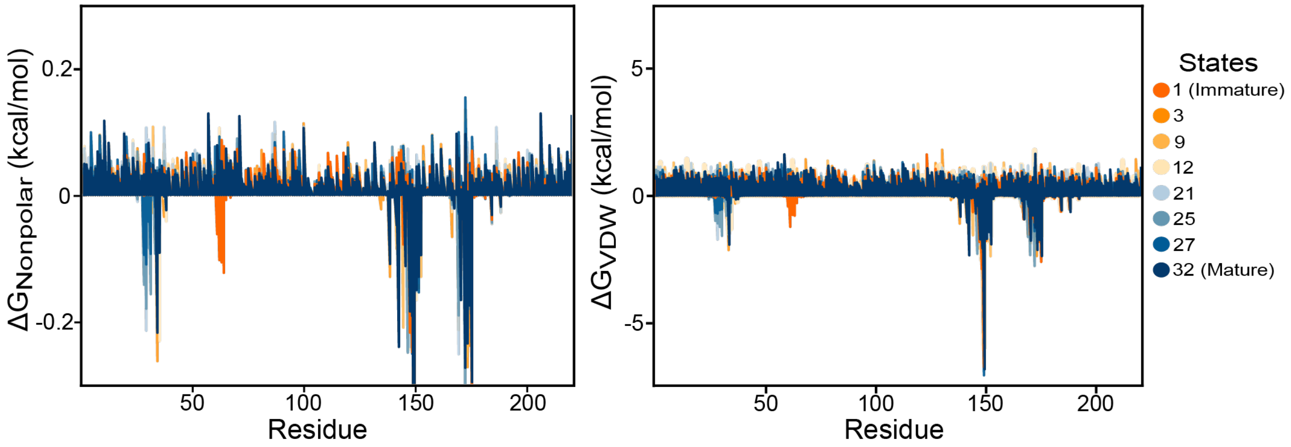

Supplementary Figure 2: The non-polar and van der Waals contribution to the free energy decomposition across distinct stages in maturation. Per-residue interaction energies are decomposed into nonpolar (left) and van der Waals (right) contributions for each residue in the HIV-1 CA monomer. Energies were computed from end-point binding free energy methods (MM/PBSA). Each line represents a distinct conformational state along the minimum free energy pathway (MFEP), from the immature state (orange) to the mature state (dark blue). Energy profiles reveal the contributions of individual residues to NTD-CTD interactions, highlighting key stabilizing interactions during CA maturation.

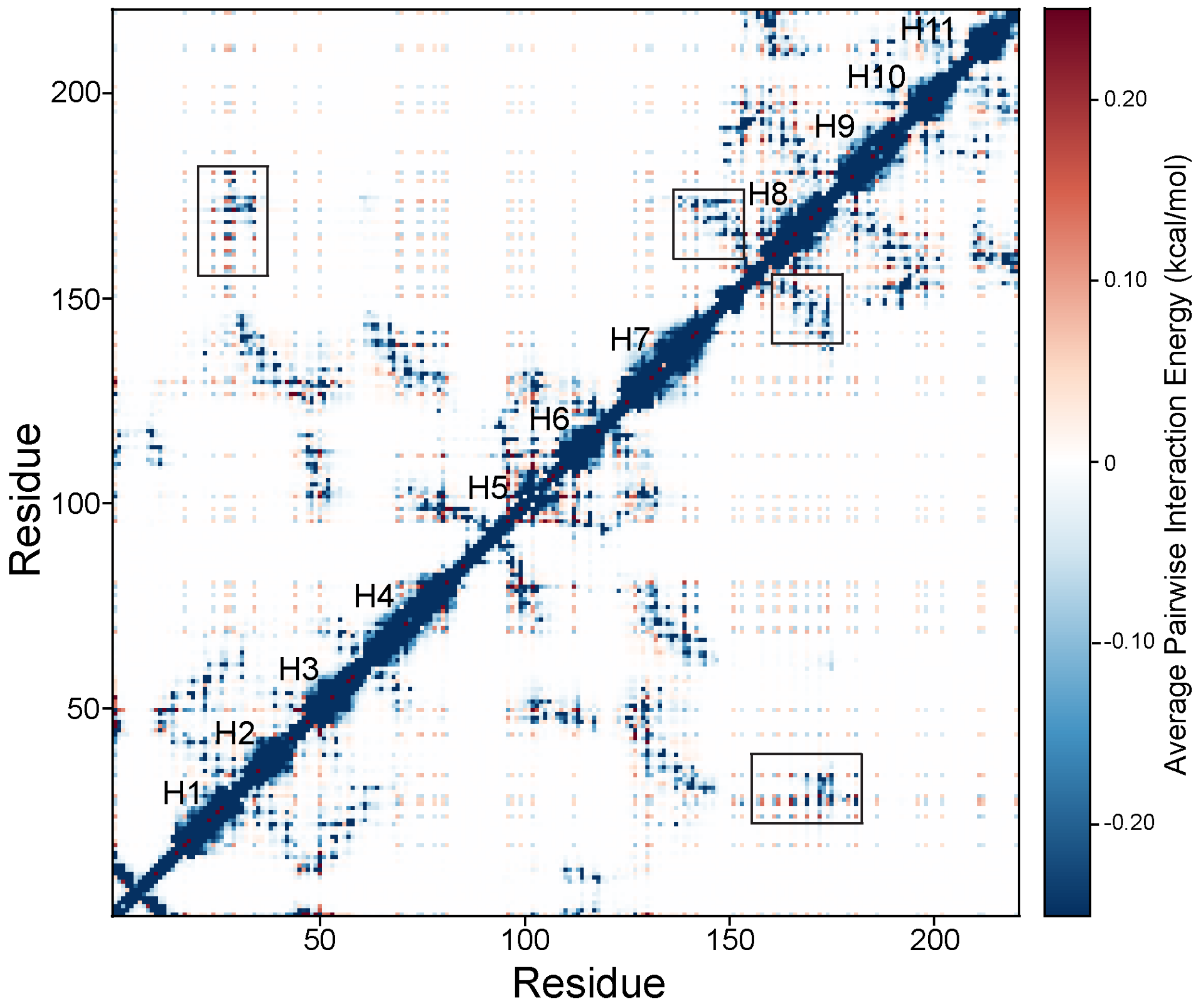

Supplementary Figure 3: Pairwise interactions between residues of the transition states along the MFEP. The heatmap shows the average pairwise interaction energy (kcal/mol) between residue pairs, computed across all 32 states along the MFEP. Blue indicates stabilizing interactions (favorable) while red denotes destabilizing interactions (unfavorable). The secondary structure interactions that form α helices are apparent on the diagonal as each residue interacts with a neighboring *i, i+4*, pattern. Off-diagonal interactions indicate higher-order tertiary interactions that stabilize the conformational state. Boxed regions correspond to pairwise residue contacts that have been identified as contributing the highest to maturation (see also Supplementary Movie 2).

Supplementary Table 1: Reproducibility checklist for MD simulations

**Reliability and reproducibility checklist for molecular dynamics simulations**<br>***All boxes must be marked YES by acceptance unless "Response not needed if No".**	**Yes**	**No**	**Response (Please state where this information can be found in the text)**
**1. Convergence of simulations and analysis**			
1a. Is an evaluation presented in the text to show that the property being measured has equilibrated in the simulations<br>(*e.g.* time-course analysis)?	☒	☐	Supplementary Figure 1 shows convergence over time.
1b. Then, is it described in the text how simulations are split into equilibration and production runs and how much data were analyzed from production runs?	☒	☐	Methods section page 16, 18-19
1c. Are there at least 3 simulations per simulation condition with statistical analysis?	☒	☐	Block averaging of multiple simulation blocks is described in Methods page 16.
1d. Is evidence provided in the text that the simulation results presented are independent of initial configuration?	☒	☐	Simulations were performed over a wide conformational space after a 2D steered MD campaign.
**2. Connection to experiments**			
2a. Are calculations provided that can connect to experiments (*e.g.* loss or gain in function from mutagenesis, binding assays, NMR chemical shifts, J-couplings, SAXS curves, interaction distances or FRET distances, structure factors, diffusion coefficients, bulk modulus and other mechanical properties, *etc*.)?	☒	☐	X-ray and cryo-EM structures are mapped onto the free energy landscape. They show good agreement with the local energy minimum.
**3. Method choice**			
3a. Do simulations contain membranes, membrane proteins, intrinsically disordered proteins, glycans, nucleic acids, polymers, or cryptic ligand binding?	☐	☒	Response not needed if **No**
3b. Is it described in the text whether the accuracy of the chosen model(s) is sufficient to address the question(s) under investigation (e.g. all-atom vs. coarse-grained models, fixed charge vs. polarizable	☒	☐	We used the most physically detailed model, including all-atom representation with explicit solvent.

force fields, implicit vs. explicit solvent or membrane, force field and water model, etc.)?			
3c. Is the timescale of the event(s) under investigation beyond the brute-force MD simulation timescale in this study that enhanced sampling methods are needed?	☒	☐	Yes
If **YES**, are the parameters and convergence criteria for the enhanced sampling method clearly stated?	☒	☐	Parameters are described in Methods, and convergence is shown in Supplementary Figure 1.
If **NO,** is the evidence provided in the text?	☐	☐	
**4. Code and reproducibility**			
4a. Is a table provided describing the system setup that includes simulation box dimensions, total number of atoms, total number of water molecules, salt concentration, lipid composition (number of molecules and type)?	☒	☐	Methods section page 13
4b. Is it described in the text what simulation and analysis software and which versions are used?	☒	☐	Methods pages 13, 17
4c. Are other parameters for the system setup described in the text, such as protonation state, type of structural restraints if applied, nonbonded cutoff, thermostat and barostat, etc.?	☒	☐	Methods pages 13, 18-19
4d. Are initial coordinate and simulation input files and a coordinate file of the final output provided as supplementary files or in a public repository?	☒	☐	Yes, on github: https://github.com/theory-and-computation/CA-maturation/
4e. Is there custom code or custom force field parameters?	☒	☐	Response not needed if **No**
If **YES**, are they provided as supplementary files or in a public repository?	☒	☐	Custom code for WHAM and analysis scripts are shared on Github.
