# Supplementary material for: Atomistic characterization of the maturation mechanisms in the HIV-1 capsid domain
Source: Nat Commun. 2026 Apr 16;17:5265. doi: 10.1038/s41467-026-71988-7 (PMC13265795; doi:10.1038/s41467-026-71988-7)
Supplement: Supplementary file 2 — Description of Additional Supplementary Files [file 41467_2026_71988_MOESM2_ESM.pdf]

Description of Additional Supplementary Files

Supplementary Movie 1. Molecular visualization and total binding free energy profiles of HIV-1 capsid along the MFEP.

Supplementary Movie 2. The molecular states of CA along the MFEP of maturation.

Supplementary Movie 3. Pairwise residue interaction heatmap of HIV-1 CA along the MFEP.
