# Supplementary material for: Atomistic characterization of the maturation mechanisms in the HIV-1 capsid domain
Source: Nat Commun. 2026 Apr 16;17:5265. doi: 10.1038/s41467-026-71988-7 (PMC13265795; doi:10.1038/s41467-026-71988-7)
Supplement: Supplementary file 7 — Transparent Peer Review file [file 41467_2026_71988_MOESM7_ESM.pdf]

nature portfolio

Peer Review File

Atomistic characterization of the maturation mechanisms in the HIV-1 capsid domain

Corresponding Author: Professor Alvin Yu

This file contains all reviewer reports in order by version, followed by all author rebuttals in order by version.

Version 0:

Reviewer comments:

Reviewer #1

(Remarks to the Author)
Capsid proteins (CA) are cleaved from the Gag lattice and must transition from their immature structure to the mature conformation that forms the infectious capsid during HIV-1 maturation. In this study, the authors used all-atom MD and enhanced sampling approaches based mostly on umbrella sampling to characterize the maturation process and quantify its underlying energetics. The free energy calculations appear to show that the immature CA conformation is intrinsically unstable and spontaneously progresses toward the mature state. Analysis of the minimum free energy path indicates that this transition occurs through a sequence of stepwise, ratcheting conformational changes; the authors identify a metastable intermediate along the pathway.

Major Comments
1. Page 4, Line 105: The authors utilize a monomer model to calculate the Minimum Free Energy Pathway (MFEP) rather than a hexamer. This model neglects the monomer-monomer interface interactions within the hexamer, which are critical for stability and conformational changes. This choice of model seems to neglect essential CA-CA interactions involved in the stability of Capsid in the physical end states, immature and mature forms. The authors should justify and elaborate on their rationale for this choice of models, and discuss how the exclusion of interfacial interactions will impact the accuracy of the calculated free energy landscape?
2. Page 5, Line 109: The text refers to "CA proteins" generally. To avoid ambiguity, please specify whether this refers to the immature or the mature form of the capsid (CA) protein in this context.
3. Page 7, Line 161: There are no error bars or uncertainty estimates provided for the reaction coordinate. Please include error analysis to demonstrate the reliability of the string method calculations in this section.
4. In Figure 2A, the distinction between conformations is unclear. Please explicitly label which colors correspond to the immature conformation and which correspond to the mature conformation.
5. Please add an explanatory note or label to Figure 2A regarding the four centers-of-mass mentioned. A reference to the Methods section for a detailed definition would also be helpful here to assist the reader in interpreting the visual data.
6. Page 9, Lines 211-220: The description of the transformation would be significantly clearer if stage numbers were explicitly added to the text. This would allow the reader to easily track the progression of the transformation alongside the figures.
7. Page 12: There appears to be no information regarding the force field used for the simulations on this page. Please provide the specific force field parameters and references to ensure reproducibility.
8. The choice of order parameters used to construct the minimum transition path from immature to mature forms is poorly explained and not well justified.

Minor Comments
1. In several figures, panels are labeled with lowercase letters (a, b, c), while the captions refer to them with uppercase letters (A, B, C). Please standardize these to uppercase (A, B, C) to ensure consistency between the images and the text.
2. Page 3, Line 79: The acronym for cryo-electron microscopy should be placed in parentheses immediately following the full term: "cryo-electron microscopy (cryo-EM)."
3. Page 4, Line 99: There is a extra period at the end of line 99.
4. Page 4, Line 103: The units used for surface area in the main text differ from those used in the figure caption. Please standardize the units across the manuscript.
5. The term "Minimum Free Energy Pathway (MFEP)" is defined multiple times. Please define it once at the first mention and

use the acronym "MFEP" consistently thereafter.
6. What do the Roman numerals denote in Figure S2? Please provide a clear definition in the figure caption.
7. Page 12, line 273: there is typo “cryo-ET”.

(Remarks on code availability)

Reviewer #2

(Remarks to the Author)
The authors present a model for the structural transitions of HIV-1 CA between the immature and mature lattices of the capsid. Using CA alone, they simulated the transition from an unstable immature conformation to the mature and more stable orientation using steering forces. The pathway between the states was analyzed to obtain a PMF through umbrella sampling and the most probable transition pathway through the string method. The authors also identified a metastable intermediate between the mature and immature orientations and analyzed the critical, stabilizing interactions along the pathway. Some interactions are briefly compared to existing literature, e.g. mutagenesis experiments. Overall, I would have liked to see some stronger experimental evidence to support the transition pathway and metastable intermediate, but this is perhaps outside of the scope of the current work. If the following comments are addressed, I would be okay with recommending this work for publication.
1. Page 3: In the introduction, the authors should elaborate briefly on the current progress of CG models for modelling the immature to mature transition process. This advancement would directly tie in with their results.
2. Page 3: the authors should also mention any previous work done in terms of modelling the capsid using enhanced sampling / path sampling methods, especially any studies using similar methods as presented here.
3. Page 5 line 113: Here the 2D order parameter used throughout the paper is introduced, but the authors should also justify their choice since order parameter selection is usually a major bottleneck for enhanced sampling. I.e., were other coordinates considered and ruled out, is there a precedent in the literature for using these coordinates and if so from which studies (or was the choice based solely on physical intuition), does the choice matter less here since there are steering forces?
4. There is evidence that BVM/lenacapavir also binds to the immature capsid and alters the maturation process (https://www.biorxiv.org/content/10.1101/2024.10.09.617473v1.full). How would lenacapavir binding effect the mechanism presented here? E.g. perhaps the metastable intermediate or the transition state would be impacted? This would be nice to mention in the discussion and/or as a future direction of study.
5. The authors also seem to be working on a CG model for the maturation process (https://www.cell.com/biophysj/abstract/S0006-3495(24)02499-8), if there is consistency between the CG maturation model and the atomistic steered MD, e.g. with a similar metastable intermediate in the CG model, this would provide some supporting evidence for the steered transition pathway. All this to say, it would be nice if the authors mentioned this in the discussion / conclusion as a point of further validation / potential next step.
6. The authors used CHARMM36m for their protein simulations and then ff14SB for their MM/PBSA calculations. Why was this inconsistent? The authors should justify this in the methods section.
7. Figure S2: It’s quite difficult to see the results here. Are there any nonpolar energy contributions at all? The plot appears blank. Can the plot be zoomed in on the Y axis, or an extra panel with a smaller Y axis could also work.

(Remarks on code availability)
The code is acceptable. More thorough README files with detailed reproduction instructions would improve the repo.

Reviewer #3

(Remarks to the Author)
This manuscript presents an extensive computational analysis of conformational transitions in the HIV-1 capsid protein (CA). The work is technically solid, clearly written, and supported by substantial sampling. The authors convincingly describe the intrinsic energy landscape connecting immature-like and mature-like conformations of an isolated CA monomer. The combination of umbrella sampling, WHAM, and the string method is carefully executed, and the identification of a monomeric metastable intermediate is a noteworthy contribution.

Major Comments
1. Scope of conclusions and interpretation of “maturation mechanism.”
The central claim that the study “uncovers the molecular mechanisms underlying CA maturation” is not fully supported by the simulations presented. All analyses are performed on a single monomer in solution, whereas CA maturation in vivo occurs within a highly constrained and multimeric environment involving the immature Gag lattice, the CA–SP1 junction, inter-hexamer cooperativity, pentamer incorporation, molecular crowding, and IP6-mediated stabilization. None of these features are modeled here. As a result, the conclusion that “the immature state is intrinsically unstable” reflects the behavior of an isolated monomer rather than the stability of the physiological immature lattice. I suggest softening this claim to:
“These results map the intrinsic monomeric energy landscape associated with NTD–CTD reorientation.”

2. Interpretation of in vitro maturation.
The manuscript interprets the rapid relaxation of the monomer toward a mature-like geometry as consistent with in vitro maturation experiments. I would recommend clarifying that, in biochemical reconstitutions, the transition from immature-like to mature-like particles primarily reflects the loss of lattice-level constraints after Gag cleavage, not intrinsic monomer instability. The immature CA conformation is well known to be stabilized by MA–CA contacts, the CA–SP1 junction, and extensive inter-hexamer interactions. A brief clarification here would avoid overinterpretation.

3. Biological context of the monomer intermediate.
The simulations very nicely characterize the intrinsic free-energy landscape of NTD–CTD reorientation, but the physiological immature conformation likely depends heavily on its multimeric and architectural context. The justification based on NMR, showing that CTD dimerization does not affect NTD–CTD rotation, does not imply that higher-order organization is irrelevant for maturation. A dedicated discussion acknowledging that the monomer landscape is only one component of the maturation process would help situate the results within the broader structural and biochemical framework, without diminishing the value of the analysis.

4. Recommendation for enhanced context and citation.
A similar computational analysis performed at the hexamer or pentamer level (e.g., Garza et al., “IP6 and PF74 affect HIV-1 capsid stability through modulation of hexamer–hexamer tilt angle preference”) may be useful to cite to situate this work relative to lattice-level studies.

Responses to Required Review Questions
• Noteworthy results:
The most significant contributions are the detailed 2D free-energy landscape, the identification of a monomeric metastable intermediate, and the residue-level energetic decomposition. These provide valuable insight into intrinsic conformational preferences of CA.
• Significance:
The work is significant as a high-resolution characterization of monomeric energetics. It complements—but does not replace—previous studies on capsid assembly, hexameric organization, and lattice stability. It will be of interest to researchers studying CA flexibility, inhibitor design, and maturation models.
• Support for conclusions:
The computational data support conclusions related to monomeric energetics. Broader conclusions about global maturation mechanisms require clarification and toning down, as noted above.
• Flaws or concerns:
There are no major flaws in the data analysis or methodology. The main issue is interpretive overreach regarding maturation. This does not prohibit publication, but should be addressed through revision.
• Methodology standard:
Yes. The methodology is sound, widely accepted, and executed with extensive sampling. The use of umbrella sampling, WHAM, and the string method is appropriate and well documented.
• Reproducibility:
Yes, the Methods provide sufficient detail for reproduction. A few clarifications (e.g., details of helix restraints, string method initial path) would further strengthen reproducibility but are not strictly required.

Minor Comments
1. Figure 1 – Unit inconsistency
Surface area is reported as “8.7 nm^2” while the rest of the manuscript uses $Å^2$. Please standardize the units.

2. Choice of structural reference
The figure 2 caption refers to PDB 5MCX, while the Methods and main text use 6BHT for the mature conformation. The rationale for choosing one structure over another, or ensuring consistency, should be clarified.

3. Movie 2
The metastable intermediate described in the text is not clearly identifiable in Movie 2. Indicating the corresponding state index or highlighting the intermediate would improve interpretability.

Overall Recommendation
This is a strong and technically rigorous study that provides valuable insight into CA monomer energetics. With clarification of scope and modest revisions to temper the mechanistic claims, the manuscript will make a meaningful contribution to the field.

(Remarks on code availability)
The inclusion of a GitHub repository containing all data, and analysis scripts is very welcome and greatly appreciated, as it substantially increases transparency and reproducibility. A more detailed README and additional in-code comments could further enhance accessibility for non-experts, but overall the availability of the full dataset is a strong asset to the manuscript

Version 1:

Reviewer comments:

Reviewer #1

(Remarks to the Author)
I think the authors could have provided a better response to my first question about the possible role of CA monomer-monomer interactions in the reorganization of CA from immature to mature form. My understanding is that there is a debate in the field as to whether the transition from the immature to mature CA lattice fundamentally involves dimeric CA units rather than the monomer. I think the authors should provide more information about this possibility to the reader. In any case, I support publication of the revised manuscript.

(Remarks on code availability)

Reviewer #2

(Remarks to the Author)
Thank you for updating your work. After reviewing the changes, the updated manuscript is now satisfactory from my end.

(Remarks on code availability)

Reviewer #3

(Remarks to the Author)
The revision and rebuttal adequately address my prior comments. The manuscript is substantially improved, and I have no remaining major concerns.

(Remarks on code availability)
The authors have adequately addressed my comments regarding the code and documentation.

Open Access This Peer Review File is licensed under a Creative Commons Attribution 4.0 International License, which permits use, sharing, adaptation, distribution and reproduction in any medium or format, as long as you give appropriate credit to the original author(s) and the source, provide a link to the Creative Commons license, and indicate if changes were made.
In cases where reviewers are anonymous, credit should be given to 'Anonymous Referee' and the source.
The images or other third party material in this Peer Review File are included in the article’s Creative Commons license, unless indicated otherwise in a credit line to the material. If material is not included in the article’s Creative Commons license and your intended use is not permitted by statutory regulation or exceeds the permitted use, you will need to obtain permission directly from the copyright holder.
To view a copy of this license, visit https://creativecommons.org/licenses/by/4.0/

We thank the reviewers and the editor for the constructive feedback. The manuscript has been extensively revised, with all major changes highlighted in **red**. The reviewers' comments are reproduced in **blue**, followed by our point-by-point responses in **black**.

Reviewer #1:

Capsid proteins (CA) are cleaved from the Gag lattice and must transition from their immature structure to the mature conformation that forms the infectious capsid during HIV-1 maturation. In this study, the authors used all-atom MD and enhanced sampling approaches based mostly on umbrella sampling to characterize the maturation process and quantify its underlying energetics. The free energy calculations appear to show that the immature CA conformation is intrinsically unstable and spontaneously progresses toward the mature state. Analysis of the minimum free energy path indicates that this transition occurs through a sequence of stepwise, ratcheting conformational changes; the authors identify a metastable intermediate along the pathway.

We thank the reviewer for an excellent review of the manuscript.

Major Comments

1. Page 4, Line 105: The authors utilize a monomer model to calculate the Minimum Free Energy Pathway (MFEP) rather than a hexamer. This model neglects the monomer-monomer interface interactions within the hexamer, which are critical for stability and conformational changes. This choice of model seems to neglect essential CA-CA interactions involved in the stability of Capsid in the physical end states, immature and mature forms. The authors should justify and elaborate on their rationale for this choice of models, and discuss how the exclusion of interfacial interactions will impact the accuracy of the calculated free energy landscape?

This is a good point. The current model was selected to capture CA conformational dynamics independent of the interface interactions. The rationale for the model is that, following proteolytic cleavage, individual CA domains can dissociate from the immature Gag lattice.

Structurally, CTD-CTD interface interactions within an immature hexamer and NTD-NTD interactions between immature hexamers are lost to form the mature capsid lattice (Fig. 1). We therefore modeled CA as a monomer to capture the intrinsic free energy landscape of the conformational changes required for maturation. By focusing on the monomer, we isolate the internal energetic barriers of the CA domains (NTD and CTD) without the effects of inter-monomer constraints to provide a landscape that dictates the protein's inherent conformations during the transition.

The calculated free energy landscape reflects the intrinsic, monomeric energetic preferences of cleaved CA domains, which exists in a dynamic “monomer-dimer” equilibrium. We selected the monomer over the dimer, because the monomer is free of physical constraints that prevent the sampling of some conformations in the dimer.

The manuscript has been updated with rationale for the choice of models in the results section (page 5, lines 108-113). Discussion on how the results extend to a multimeric context are also now included (page 12, lines 287-295).

2. Page 5, Line 109: The text refers to "CA proteins" generally. To avoid ambiguity, please specify whether this refers to the immature or the mature form of the capsid (CA) protein in this context.

References to “CA proteins” throughout the main text have been updated to specify the immature, intermediate, or mature form, depending on the context. In the NMR data referenced, it is difficult to definitively determine the conformation of CA, because the spectra include monomers and

several distinct dimer populations. We have clarified on page 5 line 119 that the data corresponds to purified CA proteins in solution.

3. Page 7, Line 161: There are no error bars or uncertainty estimates provided for the reaction coordinate. Please include error analysis to demonstrate the reliability of the string method calculations in this section.

We have included error estimates for the pathway derived from the string method in Figure 3A, shown as a shaded grey region and described in the figure caption. The highest standard deviation in the order parameter is now stated $(0.5\ \text{Å},\ 6°)$ in the results section (page 7, line 182-183).

4. In Figure 2A, the distinction between conformations is unclear. Please explicitly label which colors correspond to the immature conformation and which correspond to the mature conformation.

Figure 2a has been updated with a legend that explicitly labels the colors of the immature and mature conformations.

5. Please add an explanatory note or label to Figure 2A regarding the four centers-of-mass mentioned. A reference to the Methods section for a detailed definition would also be helpful here to assist the reader in interpreting the visual data.

Additional details on the residues used for the center-of-mass calculations have been added to the Figure 2 caption. A reference to the methods section for a more detailed definition has also been included.

.

6. Page 9, Lines 211-220: The description of the transformation would be significantly clearer if stage numbers were explicitly added to the text. This would allow the reader to easily track the progression of the transformation alongside the figures.

State numbers have been explicitly included in the text in parentheses, enabling the progression of maturation to be followed alongside more easily in conjunction with Fig. 4.

7. Page 12: There appears to be no information regarding the force field used for the simulations on this page. Please provide the specific force field parameters and references to ensure reproducibility.

We apologize for the confusion. The forcefield parameters used in the simulations are now explicitly stated on page 13, lines 311-312, and correspondingly referenced.

8. The choice of order parameters used to construct the minimum transition path from immature to mature forms is poorly explained and not well justified.

This is a good point. Order parameters are typically selected based on physical intuition with the defining characteristic that biasing forces added along such a variable can well separate between the initial and final states of interest. Based on the cryo-EM/x-ray crystallographic structures of the immature and mature states (4USN and 6BHT), we observed that the CA protein's immature and mature states are distinguished by the relative positions of the NTD and CTD, both in terms of distance between the subdomains and the angular position of the CTD relative to the NTD's principal axis. We chose the distance ξ to capture the change in distance between the NTD and

CTD, and the dihedral φ to describe the change in the CTD's rotational position about the NTD's principal axis. Steered MD simulations were used to apply biases to these two variables, and we used slow, sequential changes in each variable to steer CA from the mature state to the immature state to ensure reversibility within this collective variable space. Furthermore, the free energy landscape as seen in figure 2 and 3 is smooth, which shows that this slice of the system's phase space reasonably describes the reaction of interest. An expanded rationale and justification are now included in the methods section (page 14 lines 325–334).

Minor Comments

1. In several figures, panels are labeled with lowercase letters (a, b, c), while the captions refer to them with uppercase letters (A, B, C). Please standardize these to uppercase (A, B, C) to ensure consistency between the images and the text.

The panel labels and figure legends have been updated to use lowercase letters for consistency.

2. Page 3, Line 79: The acronym for cryo-electron microscopy should be placed in parentheses immediately following the full term: "cryo-electron microscopy (cryo-EM)."

The acronym for cryo-electron microscopy (Cryo-EM) has been added following the abbreviated word.

3. Page 4, Line 99: There is an extra period at the end of line 99.

The period has been removed from the header.

4. Page 4, Line 103: The units used for surface area in the main text differ from those used in the figure caption. Please standardize the units across the manuscript.

The units in the main text and figure legend from Fig. 1 have been standardized in units of angstroms (Å).

5. The term "Minimum Free Energy Pathway (MFEP)" is defined multiple times. Please define it once at the first mention and use the acronym "MFEP" consistently thereafter.

The term *Minimum Free Energy Pathway* is now defined once at the first mention with subsequent mentions simplified to MFEP within the main text.

6. What do the Roman numerals denote in Figure S2? Please provide a clear definition in the figure caption.

The Roman numerals have been removed and replaced with colored labels in Fig. S2 that match the state definition in Fig. 3 and 4.

7. Page 12, line 273: there is typo "cryo-ET".

The typo has been changed to cryo-EM.

Reviewer #2:

The authors present a model for the structural transitions of HIV-1 CA between the immature and mature lattices of the capsid. Using CA alone, they simulated the transition from an unstable

immature conformation to the mature and more stable orientation using steering forces. The pathway between the states was analyzed to obtain a PMF through umbrella sampling and the most probable transition pathway through the string method. The authors also identified a metastable intermediate between the mature and immature orientations and analyzed the critical, stabilizing interactions along the pathway. Some interactions are briefly compared to existing literature, e.g. mutagenesis experiments. Overall, I would have liked to see some stronger experimental evidence to support the transition pathway and metastable intermediate, but this is perhaps outside of the scope of the current work. If the following comments are addressed, I would be okay with recommending this work for publication.

We thank the reviewer for a careful and constructive evaluation of our work.

1. Page 3: In the introduction, the authors should elaborate briefly on the current progress of CG models for modelling the immature to mature transition process. This advancement would directly tie in with their results.

This is a good point. We have expanded the introduction (page 3-4, lines 81-84) to briefly summarize progress in the current state of the art in coarse-grained modeling of HIV-1 capsid protein. Existing CG models have provided insight into how the immune sensor protein TRIM5α binds to the mature HIV-1 capsid (Yu et. al., 2020, *Nature Communications*, https://doi.org/10.1038/s41467-020-15106-1), and how the nuclear pore complex facilitates import of the capsid into the nucleus (Hudait et al., 2024, *PNAS*, https://doi.org/10.1073/pnas.2313737121). However, CG models of the capsid do not currently resolve the immature-mature conformational switch. The present study provides atomic context, which can inform the development of future coarse-grained models on maturation.

2. Page 3: the authors should also mention any previous work done in terms of modelling the capsid using enhanced sampling / path sampling methods, especially any studies using similar methods as presented here.

We have revised the introduction to mention prior studies that employ enhanced-sampling methods on the HIV-1 capsid protein on page 3 lines 74-81.

3. Page 5 line 113: Here the 2D order parameter used throughout the paper is introduced, but the authors should also justify their choice since order parameter selection is usually a major bottleneck for enhanced sampling. I.e., were other coordinates considered and ruled out, is there a precedent in the literature for using these coordinates and if so from which studies (or was the choice based solely on physical intuition), does the choice matter less here since there are steering forces?

We thank the reviewer for raising this point. Several choices for the 2D order parameter were considered but ruled out based on the reversibility of the process during steered MD. Please see our response to reviewer #1, major point 8, and the methods section (page 14, lines 325-334), for a thorough explanation and updated description in the manuscript.

4. There is evidence that BVM/lenacapavir also binds to the immature capsid and alters the maturation process (https://www.biorxiv.org/content/10.1101/2024.10.09.617473v1.full). How would lenacapavir binding effect the mechanism presented here? E.g. perhaps the metastable intermediate or the transition state would be impacted? This would be nice to mention in the discussion and/or as a future direction of study.

This is an excellent point. Both lenacapavir (LEN) and bevirimat (BVM) bind to the immature capsid in the recent cryo-EM structures. BVM binds at the CA-SP1 junction in the immature lattice, stabilizing the six–helix bundle, to inhibit protease cleavage, while LEN engages the NTD interfaces across hexamers.

Notably, the inhibitor binding regions for LEN referenced (Wu *et al.*, bioRxiv, 2024; doi:10.1101/2024.10.09.617473) overlap with structural elements described in the metastable state, suggesting LEN binding could constrain CA subunits and restrict the conformational flexibility required for NTD-CTD orientation. Such inhibitor binding can be expected to increase the free energy barriers between conformational states, impeding progression along the maturation pathway.

We have referenced the study and note that extending the methods developed to examine inhibitor-CA interactions is an important direction for future study (i.e., to nominate specific CA conformations as targets for the design of maturation inhibitors) in the discussion on page 11, lines 277-284.

5. The authors also seem to be working on a CG model for the maturation process (https://www.cell.com/biophysj/abstract/S0006-3495(24)02499-8), if there is consistency between the CG maturation model and the atomistic steered MD, e.g. with a similar metastable intermediate in the CG model, this would provide some supporting evidence for the steered transition pathway. All this to say, it would be nice if the authors mentioned this in the discussion / conclusion as a point of further validation / potential next step.

We appreciate this point. Coarse-grained (CG) models of CA can be parameterized to capture the conformational rearrangements and intermediate states in CA during maturation identified by the current high-accuracy atomistic simulations. We have expanded the discussion (page 12, lines 290–295) to highlight how the results can serve as constraints for the development of future CG models of HIV virion maturation.

6. The authors used CHARMM36m for their protein simulations and then ff14SB for their MM/PBSA calculations. Why was this inconsistent? The authors should justify this in the methods section.

Coordinates for CA were extracted from the MFEP and written to PDB format. Structures were then solvated and parameterized using the AMBER ff14SB force field followed by minimization and equilibration to ensure bonded, nonbonded, and solvation terms are treated within a self-consistent energy model. We have added justification to the methods section (page 18, lines 431-435).

7. Figure S2: It's quite difficult to see the results here. Are there any nonpolar energy contributions at all? The plot appears blank. Can the plot be zoomed in on the Y axis, or an extra panel with a smaller Y axis could also work.

This is a good point. The plot has been adjusted to zoom in on the Y axis to show the nonpolar contributions.

Reviewer #2 (Remarks on code availability):
The code is acceptable. More thorough README files with detailed reproduction instructions would improve the repo.

A more detailed README and more detailed in-code comments have been included.

Reviewer #3:

This manuscript presents an extensive computational analysis of conformational transitions in the HIV-1 capsid protein (CA). The work is technically solid, clearly written, and supported by substantial sampling. The authors convincingly describe the intrinsic energy landscape connecting immature-like and mature-like conformations of an isolated CA monomer. The combination of umbrella sampling, WHAM, and the string method is carefully executed, and the identification of a monomeric metastable intermediate is a noteworthy contribution.

We thank the reviewer for the positive comments and constructive review of the manuscript.

Major Comments

1. Scope of conclusions and interpretation of “maturation mechanism.”

The central claim that the study “uncovers the molecular mechanisms underlying CA maturation” is not fully supported by the simulations presented. All analyses are performed on a single monomer in solution, whereas CA maturation in vivo occurs within a highly constrained and multimeric environment involving the immature Gag lattice, the CA–SP1 junction, inter-hexamer cooperativity, pentamer incorporation, molecular crowding, and IP6-mediated stabilization. None of these features are modeled here. As a result, the conclusion that “the immature state is intrinsically unstable” reflects the behavior of an isolated monomer rather than the stability of the physiological immature lattice. I suggest softening this claim to:

“These results map the intrinsic monomeric energy landscape associated with NTD–CTD reorientation.”

We appreciate the comments to refine the conclusions of the study. We have clarified that the immature conformation is intrinsically unstable as a monomer and softened the corresponding statements in the abstract (page 2 lines 46-47 and lines 52-54), and introduction (page 4 lines 91-92, 94, 99-100). In addition, we have added discussion on how the results may extend to the crowded macromolecular environment within virions on page 12, lines 287-295.

2. Interpretation of in vitro maturation.

The manuscript interprets the rapid relaxation of the monomer toward a mature-like geometry as consistent with in vitro maturation experiments. I would recommend clarifying that, in biochemical reconstitutions, the transition from immature-like to mature-like particles primarily reflects the loss of lattice-level constraints after Gag cleavage, not intrinsic monomer instability. The immature CA conformation is well known to be stabilized by MA–CA contacts, the CA–SP1 junction, and extensive inter-hexamer interactions. A brief clarification here would avoid overinterpretation.

This is a good point. We now clarify that in the biochemical reconstitution experiments, the transition from immature to mature particles reflects the loss of constraints imposed by contacts at the MA-CA and CA-SP1 junctions and inter-hexamer interactions. This has been incorporated in the results section (page 6, lines 143-149).

3. Biological context of the monomer intermediate.

The simulations very nicely characterize the intrinsic free-energy landscape of NTD–CTD reorientation, but the physiological immature conformation likely depends heavily on its multimeric and architectural context. The justification based on NMR, showing that CTD dimerization does not affect NTD–CTD rotation, does not imply that higher-order organization is irrelevant for maturation. A dedicated discussion acknowledging that the monomer landscape is only one component of the maturation process would help situate the results within the broader structural and biochemical framework, without diminishing the value of the analysis.

We agree that the physiological immature conformation of CA depends on multimeric interactions and the computed free energy landscape represents one component of the maturation process rather than a complete description. We have added dedicated discussion to situate the results within the broader structural context of maturation on page 12 lines 287-295.

4. Recommendation for enhanced context and citation.
A similar computational analysis performed at the hexamer or pentamer level (e.g., Garza et al., "IP6 and PF74 affect HIV-1 capsid stability through modulation of hexamer–hexamer tilt angle preference") may be useful to cite to situate this work relative to lattice-level studies.

This is a good point. We have cited the work by Garza et. al. to provide context for free energy sampling of hexamer-hexamer interactions on page 3, line 78.

Responses to Required Review Questions
• Noteworthy results:
The most significant contributions are the detailed 2D free-energy landscape, the identification of a monomeric metastable intermediate, and the residue-level energetic decomposition. These provide valuable insight into intrinsic conformational preferences of CA.
• Significance:
The work is significant as a high-resolution characterization of monomeric energetics. It complements—but does not replace—previous studies on capsid assembly, hexameric organization, and lattice stability. It will be of interest to researchers studying CA flexibility, inhibitor design, and maturation models.
• Support for conclusions:
The computational data support conclusions related to monomeric energetics. Broader conclusions about global maturation mechanisms require clarification and toning down, as noted above.
• Flaws or concerns:
There are no major flaws in the data analysis or methodology. The main issue is interpretive overreach regarding maturation. This does not prohibit publication, but should be addressed through revision.

• Methodology standard:
Yes. The methodology is sound, widely accepted, and executed with extensive sampling. The use of umbrella sampling, WHAM, and the string method is appropriate and well documented.

We thank the reviewer for the kind assessment. We have revised the manuscript to clarify the interpretation in points 1–4.

• Reproducibility:
Yes, the Methods provide sufficient detail for reproduction. A few clarifications (e.g., details of helix restraints, string method initial path) would further strengthen reproducibility but are not strictly required.

We have added additional clarification to the methods section to strengthen reproducibility including on the helical restraints and the initial path derived from the string method on page 14-15 lines 346-358, and page 17 lines 405-407.

Minor Comments
1. Figure 1 – Unit inconsistency

Surface area is reported as “8.7 nm²” while the rest of the manuscript uses Å². Please standardize the units.

The surface area in the main text and figure legend has been standardized to angstroms (Å).

2. Choice of structural reference
The figure 2 caption refers to PDB 5MCX, while the Methods and main text use 6BHT for the mature conformation. The rationale for choosing one structure over another, or ensuring consistency, should be clarified.

We thank the reviewer for pointing this out. To clarify, PDB 5MCX was used in Fig. 1 to visualize the conformations of CA in a mature lattice from cryo-EM of intact virus particles. 6BHT was used for atomic simulations owing to the high-resolution details present in the structure. There was good agreement between the variety of experimental structures of both immature and mature CA conformations in the 2D order parameter space. Clarification has been added to the main text (pg. 13 lines 303-304, and page 6, lines 137-140).

3. Movie 2
The metastable intermediate described in the text is not clearly identifiable in Movie 2. Indicating the corresponding state index or highlighting the intermediate would improve interpretability.

Supplementary Movie 2 has been updated to label the metastable intermediate along with the corresponding state index.

Overall Recommendation
This is a strong and technically rigorous study that provides valuable insight into CA monomer energetics. With clarification of scope and modest revisions to temper the mechanistic claims, the manuscript will make a meaningful contribution to the field.

We hope that the revised manuscript, with clarified scope and requested clarifications, addresses the concerns and strengthens the study.

Reviewer #3 (Remarks on code availability):
The inclusion of a GitHub repository containing all data, and analysis scripts is very welcome and greatly appreciated, as it substantially increases transparency and reproducibility. A more detailed README and additional in-code comments could further enhance accessibility for non-experts, but overall the availability of the full dataset is a strong asset to the manuscript

A more detailed README including instructions and guide and additional detailed in-code comments have been added to the GitHub repository.

The manuscript has been revised, with all content changes highlighted in **red**. The reviewers' comments are reproduced in **blue**, followed by our point-by-point responses in **black**.

Reviewer #1 (Remarks to the Author):

I think the authors could have provided a better response to my first question about the possible role of CA monomer-monomer interactions in the reorganization of CA from immature to mature form. My understanding is that there is a debate in the field as to whether the transition from the immature to mature CA lattice fundamentally involves dimeric CA units rather than the monomer. I think the authors should provide more information about this possibility to the reader. In any case, I support publication of the revised manuscript.

We appreciate the continued feedback. We have expanded the discussion and rationale of the model to provide additional context to the reader on the involvement of dimers, and higher-order oligomers in CA maturation on pg. 5 lines 107–114 and 123–124.

We thank the reviewer for the constructive review of the manuscript and hope it has been satisfactorily improved.

Reviewer #2 (Remarks to the Author):

Thank you for updating your work. After reviewing the changes, the updated manuscript is now satisfactory from my end.

We thank the reviewer for the feedback and constructive review.

Reviewer #3 (Remarks to the Author):

The revision and rebuttal adequately address my prior comments. The manuscript is substantially improved, and I have no remaining major concerns.

Reviewer #3 (Remarks on code availability):

The authors have adequately addressed my comments regarding the code and documentation.

We thank the reviewer for the feedback and constructive review.
